# Differential Expression of Salivary Proteins between Susceptible and Insecticide-Resistant Mosquitoes of *Culex quinquefasciatus*

**DOI:** 10.1371/journal.pone.0017496

**Published:** 2011-03-23

**Authors:** Innocent Djegbe, Sylvie Cornelie, Marie Rossignol, Edith Demettre, Martial Seveno, Franck Remoue, Vincent Corbel

**Affiliations:** 1 Institut de Recherche pour le Développement (IRD), Maladies Infectieuses et Vecteurs, Ecologie, Génétique, Evolution et Contrôle (MIVEGEC), UM1-CNRS 5290-IRD 224, Centre de Recherche Entomologique de Cotonou (CREC), Cotonou, Bénin; 2 Institut de Recherche pour le Développement (IRD), MIVEGEC, UM1-CNRS 5290-IRD 224, Laboratoire de lutte contre les Insectes Nuisibles (LIN), Centre IRD, Montpellier, France; 3 Plate-forme de Protéomique Fonctionnelle, IGF, CNRS- UMR 5203, INSERM U661, Université Montpellier I et II, Montpellier, France; University of Western Cape, South Africa

## Abstract

**Background:**

The *Culex quinquefasciatus* mosquito, a major pest and vector of filariasis and arboviruses in the tropics, has developed multiple resistance mechanisms to the main insecticide classes currently available in public health. Among them, the insensitive acetylcholinesterase (a*ce-1^R^* allele) is widespread worldwide and confers cross-resistance to organophosphates and carbamates. Fortunately, in an insecticide-free environment, this mutation is associated with a severe genetic cost that can affect various life history traits. Salivary proteins are directly involved in human-vector contact during biting and therefore play a key role in pathogen transmission.

**Methods and Results:**

An original proteomic approach combining 2D-electrophoresis and mass spectrometry was adopted to compare the salivary expression profiles of two strains of *C. quinquefasciatus* with the same genetic background but carrying either the a*ce-1^R^* resistance allele or not (wild type). Four salivary proteins were differentially expressed (>2 fold, P<0.05) in susceptible (SLAB) and resistant (SR) mosquito strains. Protein identification indicated that the D7 long form, a major salivary protein involved in blood feeding success, presented lower expression in the resistant strain than the susceptible strain. In contrast, three other proteins, including metabolic enzymes (endoplasmin, triosephosphate isomerase) were significantly over-expressed in the salivary gland of a*ce-1^R^* resistant mosquitoes. A catalogue of 67 salivary proteins of *C. quinquefasciatus* sialotranscriptome was also identified and described.

**Conclusion:**

The “resistance”-dependent expression of salivary proteins in mosquitoes may have considerable impact on biting behaviour and hence on the capacity to transmit parasites/viruses to humans. The behaviour of susceptible and insecticide-resistant mosquitoes in the presence of vertebrate hosts and its impact on pathogen transmission urgently requires further investigation.

**Data Deposition:**

All proteomic data will be deposited at PRIDE (http://www.ebi.ac.uk/pride/).

## Introduction

*Culex pipiens quinquefasciatus* is an important vector of Bancroftian filariasis and arboviruses worldwide and represents the main mosquito nuisance in urban environments [1], [2]. Vector control against this mosquito species relies essentially on environmental sanitation and the use of insecticides in polluted breeding habitats [3]. Unfortunately, resistance to insecticides in *C. quinquefasciatus* mosquitoes emerged more than 25 years ago in Africa, America and Europe and this resistance is frequently due to a loss of sensitivity of the insect's acetylcholinesterase enzyme to organophosphates and carbamates [4]. Two amino acid substitutions (i.e. F290V, G119S) were found to play a role in resistance [5] but the G119S resistant allele (named *ace-1^R^*) was shown to be widespread in *C. quinquefasciatus* natural populations [6], [7], [8], [9]. Fortunately, although insecticide resistance alleles afford a selective advantage in the presence of insecticide, they can constitute a handicap in an insecticide-free environment [10], [11]. Previous studies reported that *ace-1^R^* alleles have a strong genetic cost that can induce important behavioural and physiological changes in insects [12], [13]. In *C. quinquefasciatus*, *ace-1* alleles coding for a modified AChE1 were associated with a longer development time, lower emergence rates and shorter wing length than in their susceptible counterparts [14], [15]. Other studies showed that the resistant larvae of *C. quinquefasciatus* were also less able to escape predation [16] and adult males were less competitive for mating than wild-type susceptible males [17]. The *ace-1^R^* allele is known to be the most costly of resistance genes because it interferes with the general functioning of the central nervous system throughout a mosquito's life and adversely modifies behavioural traits [18], [19].

Far less information is available about the impact of insecticide resistance alleles affecting other traits such as host seeking and blood feeding behaviour. Caroll *et al.* [20] first demonstrated that insecticide resistance in *C. quinquefasciatus* mosquitoes could interfere with the development of parasites, i.e. organophosphate-resistant mosquitoes were less likely to transmit filariasis than their insecticide-susceptible counterparts [20]. Before taking a blood meal, mosquito females inject several salivary substances into the host skin to counteract the haemostatic reaction induced by the bite [21]. The main functions of the saliva are powerful anti-coagulation, vasodilatation and platelet aggregation inhibition [22] that favour the blood feeding success. Mosquito salivary proteins then play a major role in host-vector interaction and can also interfere with pathogen transmission [23] including that of arboviral viruses [24] and parasites [25]. Here, an original approach by proteomic technology combining 2D-electrophoresis (2DE) and mass-spectrometry (MS) was used to compare the salivary expression profile of two strains of *C. quinquefasciatus* having same genetic background but either carrying the a*ce-1^R^* resistance allele or not (wild type). The hypothesis is that the genetic cost associated with the *ace.1^R^* allele may modulate the expression of salivary proteins in *C. quinquefasciatus* salivary glands. In our study, differences between strains could be directly attributed to the expression of resistance alleles in a standard genetic environment. An updated list of salivary proteins from *C. quinquefasciatus* sialotranscriptome is also provided.

## Materials and Methods

### *Culex* mosquito strains

Two strains of *C. quinquefasciatus* were used; SLAB and SR. They all share the same genetic background and only differ in their genotype at the *ace-1* locus [15]. SLAB, the insecticide-susceptible reference strain [26], is homozygous for susceptible alleles at the a*ce-1* locus. SR is homozygous for the resistant allele a*ce-1^R^* which was introgressed into the genome of SLAB through 14 repeated generations of backcrossing [17].

### Preparation of salivary gland extracts

Unfed mosquitoes of the SLAB and SR strains, 7 days old, were first sedated with CO_2_. The salivary glands were dissected and then transferred to rehydratation buffer and stored at −80°C before use. A total of 30 batches of 30 pairs of salivary glands from *C. quinquefasciatus* females were obtained per strain. The salivary glands were lysed in liquid nitrogen and homogenates were then centrifuged for 30 min at 30,000 g at 4°C. The supernatants, named Salivary Gland Extracts (SGE) containing soluble salivary proteins, were subjected to two-dimensional electrophoresis (2DE). All reagents used for 2DE were from the Plus One range (GE Healthcare Biosciences, Uppsala, Sweden).

### Two-dimensional electrophoresis

2DE was carried out with 22 µg of *C. quinquesfasciatus* SGE on 11 cm immobiline™ dryStrips pH 3–11 non linear (NL) (GE Healthcare, Germany). Strips were rehydrated for 10–20 h at 20°C with protein samples made up to 170 μl by adding IEF buffer (7 M urea, 2 M thiourea, 4% CHAPS, 0.2% tergitol, 0.8% IPG buffer, and 1,2% DeStreak reagent). Running conditions were: temperature 20°C; current 50 μA per strip; 300 V (gradient) for 5 min; 300 V (step) for 30 min, 5000 V (gradient) for 3 h, and then 5000 V steps up to 60000 Vh. The second dimension was carried out on 10–20% SDS-PAGE gels (Biorad, Marnes la Coquette, France) at 70 V for 15 min and then 200 V until the bromophenol blue front had reached the end of the gel. The gels were fixed 20 minutes in 50% ethanol/5% acetic acid solution, and then 10 min in 50% ethanol solution, washed four times in MilliQ water. Finally, gels were stained with colloidal coomassie blue (Fermentas, Saint-Remy les Chevreuse, France) overnight and washed twice in milliQ water. Gels were scanned with a EPSON Perfection pro V750. All gel images were acquired at 16 bits resolution under non saturating conditions. 2DE images were analyzed using *Same Spots*™ Software 3.3 (Nonlinear Dynamics). Statistical analysis and protein quantification were carried out using the same software. First, PCA analysis was performed to verify that the gels from both strains (SLAB and SR) were distributed in two distinct groups. Secondly, statistical analysis was done by an ANOVA test (P<0.05) for all spots in both groups. About 20 spots reached the threshold of significant differential expression between both strains, and a second statistical analysis taking into account possible false positives was then performed with a cut-off of 2 fold in either direction (up and down-expression) and with P<0.05 and power >0.8. The q value represents therefore the P value adjusted by the False Discovery Rate (FDR). Details are indicated in: http://www.nonlinear.com/support/progenesis/samespots/faq/pq-values.aspx). Protein spots of each strain were digested by trypsin and identified by mass-spectrometry.

### Identification of salivary proteins by mass-spectrometry

#### Trypsin digestion

Enzymatic in-gel digestion was performed automatically (Tecan freedom evo® proteomics) according to the Shevchenko modified protocol [27].

Briefly, protein spots were digested using 150 ng of trypsin, peptide extraction was performed using 5 sonication cycles of 2 min each and peptides were concentrated 1 hour at 50°C in a heat block. Peptide samples were automatically spotted (Tecan freedom evo® proteomics). For this step, 0.5 µl of sample peptide and 0.5 µl of alpha-cyano-4-hydroxy-*trans*-cinnamic acid (a saturated solution prepared in acetonitrile/trifluoroacetic acid, 50 ∶ 0.1%, vortexed, sonicated 30 s and microcentrifuged 30 s with a 1/3 dilution of the supernatant used as the matrix) were deposited on a 384-well MALDI anchorship target using the dry-droplet procedure [28] and air dried at room temperature. Peptide samples were then desalted using a 10 mM phosphate buffer and dried again at room temperature. Mass spectrometry was then performed on both SLAB and SR strains of *C. quinquefasciatus*.

#### MALDI-TOF MS analysis

Analyses were performed using an UltraFlex MALDI TOF-TOF mass spectrometer (Bruker Daltonics, Bremen, Germany) in the reflectron mode with a 26 kV accelerating voltage and a 50 ns delayed extraction. Mass spectra were acquired in automatic mode using the AutoXecute™ module of Flexcontrol™ (Bruker Daltonics) (laser power ranged from 40 to 50%, 600 shots). Spectra were analyzed using FlexAnalysis™ software (Bruker Daltonics) and calibrated internally with the autoproteolysis peptides of trypsin (m/z: 842.51; 1045.56; 2211.10). Peptides were selected in the mass range of 900–3000 Da.

Peptide mass fingerprint identification of proteins was performed by searching against the Insecta entries of either SwissProt or TrEMBL databases (http://www.expasy.ch) using the Mascot v 2.2 algorithm (http://www.matrixscience.com) as previously described [29] Mascot scores higher than 65 were considered as significant (P<0.05) for the SwissProt and TrEMBL databases

#### Nano LC-MS/MS analysis

Protein samples that could not be identified by MALDI-TOF MS analysis were subjected to nano LC ESI MS/MS analysis with a QTOF or a LTQ Orbitrap XL. Samples were dehydrated in a vacuum centrifuge, solubilized in 1 µl of 0.1% formic acid-2% acetonitrile and analyzed online on a ESI quadrupole time-of-flight (Q-TOF) mass spectrometer (QSTAR Pulsar-*i*, Applied Biosystems, Foster City, CA) or a ESI LTQ Orbitrap mass spectrometer (LTQ Orbitrap XL, Thermo Fisher Scientific) respectively, coupled with an Ultimate 3000 HPLC (Dionex, Amsterdam, Netherlands). Details are given in Table S1.

Regarding the protein identification, all MS/MS spectra were searched against the Insecta entries of either SwissProt or TrEMBL databases by using the Mascot v 2.2 algorithm (MA; Matrix Science Inc.) with trypsin enzyme specificity and one missed trypsin cleavage. With nano LC ESI LTQ Orbitrap XL analysis, the data submission was performed using ProteomeDiscoverer v 1.0 (Thermo Fisher Scientific). Peptides with scores greater than the identity score (P<0.05) were considered significant. All spectra were manually validated for proteins identified with less than three different peptides.

## Results

### Differential expression profile of sialome between susceptible and a*ce-1^R^* resistant *Culex* mosquitoes

Differential sialome expression between susceptible (SLAB) and resistant (SR) strains of *C. quinquefasciatus* was assessed by comparing 2D-electrophoresis gels.

**Figure 1:**
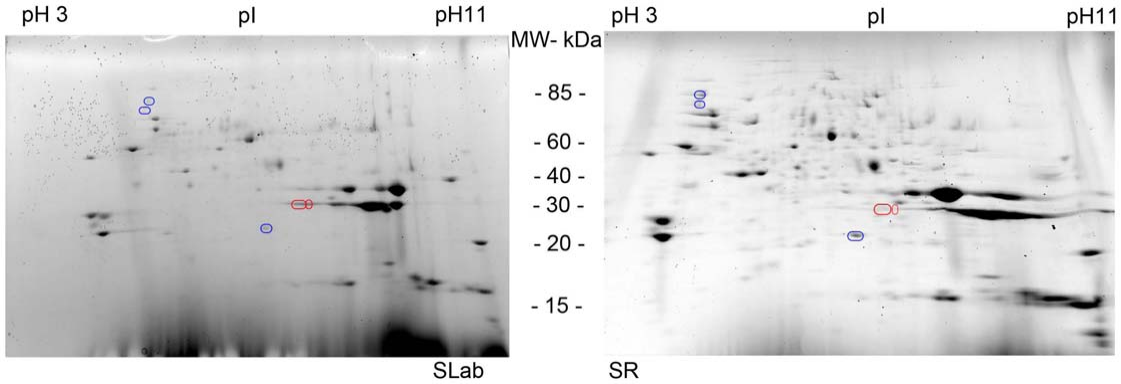
2D electrophoresis profile (SDS-Page) of *C. quinquefasciatus* salivary gland proteins. Salivary gland extracts (SGE) from the susceptible SLAB strain (left panel) and the a*ce.1* resistant SR strain (right panel) are shown. Concentrations were measured according to the Bradford method; 729 µg/ml for SLAB strain and 816 µg/ml for SR strain. Protein spots showing increased or decreased expression in the resistant strain are circled in blue and red, respectively.

**Figure 2:**
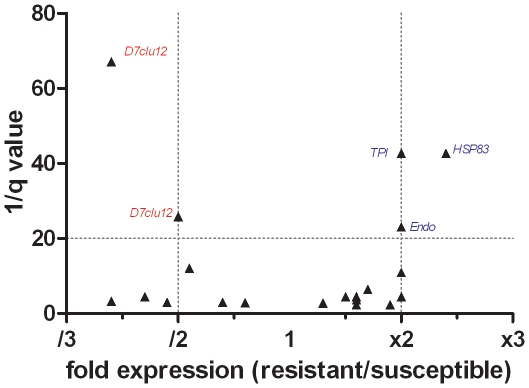
Differential salivary protein expression between susceptible and *ace.1* resistant *C. quinquefasciatus*. Salivary gland extractions were carried out using 7-day old unfed females. Differences in protein expression are indicated as a function of both expression ratio (resistant/susceptible) and significance ratio (q value). Vertical lines indicate two-fold differential expression in either direction. Horizontal lines indicate the significance threshold (1/q<20) or q<0.05) according to Same Spot analysis. Proteins showing more than 2.0 fold expression and a significant 1/q value are named.

In overall, 14 gels were obtained for each strain (SLAB and SR) and 322 spots (excluding artefacts) were detected (Figure 1). Six of 14 gels were excluded per strain because of unreliable spot focalization. On the remaining gels, Principal Component Analysis showed that spot profiles significantly differed between the resistant and susceptible strains (variance >46%, data not shown). The first set of ANOVA analysis detected 20 spots showing differential expression between the SLAB and SR strains (Figure 2) but after adjustment using the FDR approach, only 5 of these showed significant differential expression (q<0.05, Power >0.8) (Figure 2, Table S1). Two spots (in red) showed two-fold under-expression in resistant strain SR compared to the SLAB strain whereas three other spots (in blue) showed two-fold over-expression in the resistant strain.

### Identification of salivary gland proteins by mass spectrometry

**Figure 3:**
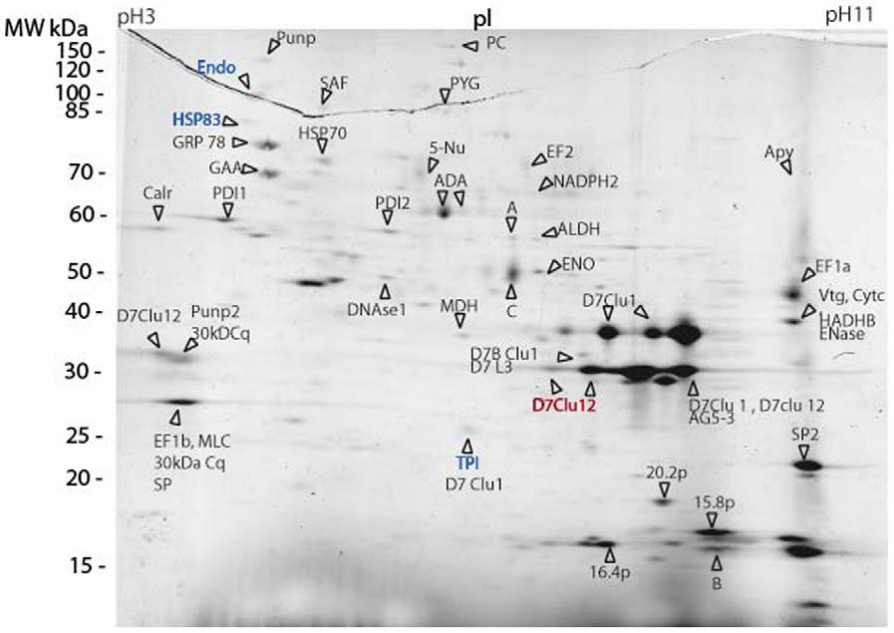
Identification of *C. quinquefasciatus* salivary proteins by mass spectrometry. Proteins are indicated in a 2D-gels for susceptible and resistant (*ace.1^R^*) mosquitoes with spots named according to Table 1. A total of 89 spots were analyzed and a catalogue of 52 salivary proteins is shown. Proteins which are more highly expressed in the resistant (SR) strain than SLAB are coded in blue whereas those under-expressed are coded in red. Spots were excised manually and then digested and extracted with TECAN EVO or manually. Proteins were identified by MALDI-TOF and if the identification was ambiguous, LC-MS/ MS was performed.

The second step was to characterize the sialome of *C. quinquefasciatus* using both MALDI-TOF and LC-MS/MS mass-spectrometry. A total of 89 spots were analyzed by MA and a catalogue of 52 salivary proteins were described (Figure 3). The identified proteins could be split into three functional classes: “salivary” products, housekeeping products and products of “unknown” function (Table 1). Of the identified proteins, 43% were secreted and 57% were intracellular.

**Table 1:** Identification and classification of *C. quinquefasciatus* salivary proteins.

Abbreviation	Protein Identification	function	Accession Number	Nominal Mass (kDa)	Pi	Sequence Coverage %	MS source	Spot ID
NADPH2	Glutamate semialdehyde dehydrogenase – Culex quinquefasciatus	amine metabolism	B0X119_CULQU	86226	6,7	36	MALDI TOF	545–606
30 kDa Cq	30 kDa salivary gland allergen Aed a 3 – Culex quinquefasciatus	antigen V family	B0W7N1_CULQU	27712	4,57	5	LC-MS/MS QTOF	521–565 522
AG5-3	Salivary secreted antigen-5 AG5-3 – Culex quinquefasciatus	antigen V family	B0XGB4_CULQU	29925	7,14	4	LC-MS/MS QTOF	558
ADA	Salivary adenosine deaminase – Culex quinquefasciatus	blood feeding	Q95WT8_CULQU	57849	5,86	32	MALDI TOF	541–544–573
Apy	Apyrase – Culex quinquefasciatus	blood feeding	B0WUA1_CULQU	59481	7,21	21	MALDI TOF	597
D7 Clu 1	Long form D7clu1 salivary protein – Culex quinquefasciatus	blood feeding	Q95V93_CULQU	35750	7,45	41	MALDI TOF	553–555–578–580
***D7 Clu 12***	***Long form D7clu12 salivary protein – Culex quinquefasciatus***	***blood feeding***	***Q95V92_CULQU***	***36537***	***7,5***	***51***	***MALDI TOF***	***581–558–523–530–564–587–588***
C = D7-L3	Salivary long D7 protein 3 – Culex quinquefasciatus	blood feeding	B0X6Z3_CULQU	27332	7,53	15	LTQ-Orbitrap	594 = C
D7-L3	Salivary long D7 protein 3 – Culex quinquefasciatus	blood feeding	Q6TRZ6_CULQU	39205	6,67	29	MALDI TOF	579
5-Nu	Salivary apyrase; 5′ nucleotidase – Culex quinquefasciatus	blood feeding	B0XHG2_CULQU	62553	5,47	21	MALDI TOF	602
E3	Dihydrolipoyl dehydrogenase, mitochondrial – Culex quinquefasciatus	citric acid cycle, glycolyis	B0X2P1_CULQU	37768	5,53	38	MALDI TOF	547 = A, 549,607
ALDH	Aldehyde dehydrogenase, mitochondrial – Culex quinquefasciatus		B0WKSO_CULQU	57133	7,64	23		
E2	Dihydrolipoamide acetyltransferase component of pyruvate dehydrogenase Culex quinquefasciatus		B0XAPO_CULQU	54962	9	22		
SCSb	Succinyl-coa synthetase beta chain – Culex quinquefasciatus	citric acid cycle	B0WFW7_CULQU	48729	7,1	16	LTQ-Orbitrap	594 = C
AcoA	Acyl-coa dehydrogenase – Culex quinquefasciatus	citric acid cycle	B0WLI6_CULQU	46057	8,37	10	LTQ-Orbitrap	594 = C
MLC	Myosin light chain 2 – Culex quinquefasciatus	cytosqueletton	B0XDR8_CULQU	22838	4,65	29	LC-MS/MS QTOF	521–565
VTG	Vitellogenin – Culex quinquefasciatus	endocrinal pathway	B0X7X6_CULQU	42128	8,36	11	MALDI TOF	585
HADHB	3-ketoacyl-CoA thiolase – Culex quinquefasciatus	fatty acid oxydation	B0W5M7_CULQU	41730	8,59	2	MALDI TOF	585
MDH	Malate dehydrogenase – Culex quinquefasciatus	gluconeogenesis	B0W5T5_CULQU	35315	6,15	32	MALDI TOF	574–542
PC	Pyruvate carboxylase, mitochondrial – Culex quinquefasciatus	gluconeogenesis	B0W649_CULQU	133344	6,59	34	MALDI TOF	540
ENO	Enolase – Culex quinquefasciatus	glycolysis	B0W1N4_CULQU	46908	6,29	19	MALDI TOF	550
***TPI***	***Triosephosphate isomerase – Culex quinquefasciatus***	***glycolysis***	***B0W5W4_CULQU***	***24075***	***6***	***12***	***LC-MS/MS QTOF***	***543***
C = CAP	Adenylyl cyclase-associated protein – Culex quinquefasciatus	inositol cycle	B0W727_CULQU	68046	5,32	10	LTQ-Orbitrap	594 = C
DNAse 1	Deoxyribonuclease I – Culex quinquefasciatus	nucleic acid metabolism	B0W7Z5_CULQU	46863	5,61	20	MALDI TOF	593
Enase	CULQU Salivary endonuclease – Culex quinquefasciatus	nucleic acid metabolism	B0WQ10_CULQU	42721	9,28	3	MALDI TOF	585
CALR	Calreticulin – Culex quinquefasciatus	protein folding	B0WJE0_CULQU	46874	4,37	51	MALDI TOF	596– 524
PDI-1	Disulfide isomerase – Culex quinquefasciatus	protein folding	B0X3M7_CULQU	55733	4,82	46	MALDI TOF	525–566
PDI- 2	Disulfide isomerase – Culex quinquefasciatus	protein folding	B0X904_CULQU	54158	5,9	40	MALDI TOF	534–572–595
***Endo***	***Endoplasmin – Culex quinquefasciatus***	***protein folding***	***B0W5Z4_CULQU***	***91045***	***4,9***	***40***	***MALDI TOF***	***592–526–591***
HSP 70	Heat shock 70 kDa protein cognate 4 – Culex quinquefasciatus	protein folding- stress response	B0WP93_CULQU	71787	5,36	41	MALDI TOF	532–609
***HSP 83***	***Heat shock protein 83 – Culex quinquefasciatus***	***protein folding- stress response***	***B0WX04_CULQU***	***81938***	***4,9***	***37***	***MALDI TOF***	***590***
EF1a	Elongation factor 1-alpha 1 – Culex quinquefasciatus	protein synthesis	B0WQ61_CULQU	52582	9,23	29	MALDI TOF	559
EF1B	Elongation factor 1-beta – Culex quinquefasciatus	protein synthesis	B0WF81_CULQU	24630	4,57	9	LC- MS/MS QTOF	521–565
EF 2	Elongation factor 2 – Culex quinquefasciatus	protein synthesis	B0W238_CULQU	115611	6,29	19	MALDI TOF	546
SAF	Spermatogenesis associated factor – Culex quinquefasciatus	related to ATP-binding proteins	B0WC89_CULQU	88819	5,23	18	MALDI TOF	604
GRP 78	78 kDa glucose-regulated protein - Culex quinquefasciatus	related to HSP 70 family	B0W934_CULQU	72377	5,07	37	MALDI TOF	605
Punp	Putative uncharacterized protein- Culex quinquefasciatus	related to HSP family	B0WKR8_CULQU	103860	4,93	26	MALDI TOF	600
CytC	Ubiquinol-cytochrome c reductase complex core protein - Culex quinquefasciatus	respiratory reaction	B0WXM0_CULQU	45651	9,02	11	MALDI TOF	585
C = Hrb27c	Heterogeneous nuclear ribonucleoprotein 27C OS - Culex quinquefasciatus	ribonucleosome component	B0X7P8_CULQU	42660	6,54	18	LTQ-Orbitrap	594 = C
GAA	Alpha-glucosidase - Culex quinquefasciatus	sugar feeding	B0XAA1_CULQU	66690	5,08	36	MALDI TOF	567–610
PYG	Glycogen phosphorylase - Culex quinquefasciatus	sugar metabolism	B0WCF2_CULQU	97096	5,96	22	MALDI TOF	539
15.8p	Putative 15.8 kDa salivary peptide - Culex quinquefasciatus	unknown	Q6TRX5_CULQU	17826	8,75	39	MALDI TOF	598
16.4p	16.4 kDa salivary peptide - Culex quinquefasciatus	unknown	Q6TS05_CULQU	18312	6,82	37	MALDI TOF	556–582
20.2p	20.2 kDa salivary peptide - Culex quinquefasciatus	unknown	B0WZL9_CULQU	23264	8,65	39	MALDI TOF	557–583–599
16p	16 kDa salivary peptide - Culex quinquefasciatus	unknown	Q6TRZ5_CULQU	18301	9,02	15	LC-MS/MS QTOF	584 = B
16.8p	Putative 16.8 kDa salivary protein - Culex quinquefasciatus	unknown	Q6TRY2_CULQU	18773	8,66	7		
16.8p1	Putative 16.8 kDa salivary peptide - Culex quinquefasciatus	unknown	Q6TS03_CULQU	19267	8,78	6		
H2BF	Histone H2B.a/g/k - Culex quinquefasciatus	DNA structure	B0WG57_CULQU	5744	10,1	16		
13.1p	Putative 13.1 kDa salivary protein - Culex quinquefasciatus	unknown	Q6TS27_CULQU	15209	9,47	8		
Punp1	Putative uncharacterized protein - Culex quinquefasciatus	unknown	B0X1P5_CULQU	27764	4,72	23	LC-MS/MS QTOF	522
SP	Salivary protein - Culex quinquefasciatus	unknown	B0W4E3_CULQU	24461	4,54	7	LC-MS/MS QTOF	521–565
SP2	Salivary protein - Culex quinquefasciatus	unknown	Q6TS09_CULQU	23785	9,18	67	MALDI TOF	562

Database searches used the MASCOT program and SwissProt/TrEMBL databases. Molecular mass, pI and sequence coverage are shown. All the MASCOT scores are p>65. Proteins are divided into housekeeping products (proteins expressed routinely in the cell) salivary proteins, including proteins involved in blood and/or sugar feeding, and unknown proteins for which no function has been defined.

Among the “salivary products” that are specifically expressed in the mosquito salivary glands, two spots corresponding to a unique protein named the D7 long form cluster12 protein showed two-fold lower expression in the resistant strain SR compared to the SLAB strain (Figure 2). The D7 family is widely distributed in mosquito salivary glands and plays a key role in blood feeding success [30], [31]. Other salivary proteins were identified including apyrase, 5′nucleotidase, antigen 5 family (30 kDa Cq, AG5-3) and adenosine deaminase, which is believed to be involved in blood feeding (Figure 3, Table 1).

Of the housekeeping products, which include many proteins involved in metabolism, three proteins showed two-fold higher expression in the resistant strain (Figure 2). These proteins were identified as endoplasmin, triosephosphate isomerase and a heat shock protein (HSP83). These proteins are known to be involved in protein folding, glycolysis and stress response, respectively. Among other housekeeping proteins, the salivary endonuclease which belongs to the hydrolase family was also identified (Table 1): this protein is believed to reduce local blood viscosity at the bite site to enhance the feeding process [31].

Among the “unknown” products, no significant difference in protein expression was noted between the susceptible and resistant strains (P>0.05). Most of secreted salivary peptides identified belonged to the cystein and tryptophan rich protein family of the *Culex* genus (CWRP- peptide 15.8p, 16.4p, 16p, 16.8p, 16.8p1, and 13.1p). As previously described, this family is highly expressed in the salivary glands of *C. quinquesfacsiatus* [31]: it has been proposed that these may antagonise serotonin and histamine but their role in the host-vector relationships needs further investigation.

## Discussion

In the present study, we compared the expression of salivary proteins of two mosquito strains of *C. quinquefasciatus* with same genetic background but carrying either the a*ce-1^R^* resistance allele or not.

Our results showed that four proteins were differentially expressed in the *C. quinquefasciatus* sialome between the resistant (*ace.1* ^R^) and the susceptible strain (q<0.05 and Power >0.8). To our knowledge, this is the first evidence that an insecticide-resistance gene can modulate the expression of salivary proteins in Diptera. Among these 4 proteins, the D7 long form salivary protein, which is secreted by mosquitoes during the blood meal [32], was significantly underexpressed in the resistant strain compared to the susceptible strain. This protein is present in all haematophagous diptera and may play a major role in blood feeding success. In mosquitoes, the D7 long form protein is an important component of the salivary glands and belongs to the super family of odorant binding proteins [30]. Although their function in blood feeding remains unclear, it has been suggested that this protein could sequester and inhibit biogenic amines (serotonin, histamine) involved in inflammation and pain during the bite [31]. The consequences of lower expression of the D7 protein are currently unknown but it speculates that its down-expression may compromise *C. quinquefasciatus* blood feeding and could therefore affect the transmission of pathogens to humans. In addition, major salivary proteins - including the apyrase and D7 proteins - are known to be significantly down-regulated in infected *Anopheles* compared to uninfected mosquitoes [33], [34]. These findings emphasise the need for further experiments to assess the impact of pathogen infection on salivary protein expression in both susceptible and *Ace.1^R^* resistant mosquitoes.

Conversely, three other proteins, namely endoplasmin, triosephosphate isomerase and heat shock protein (HSP) were significantly over-expressed in the salivary glands of *Ace.1* resistant mosquitoes. Triosephosphate isomerase is an enzyme involved in glycolysis which takes place in the cytosol of cells. Over-expression of this enzyme suggests modulation of the metabolic activity of the salivary gland in the resistant strain. The HSP was the most stress-responsive protein in diptera and has also been identified in humans and rodents [35]. Over-expression of this protein may be explained by the fact that salivary gland tissues may be modified in the presence of *Ace.1* ^R^ allele, e.g. by stress. Endoplasmin is produced by the endoplasmic reticulum and acts as molecular chaperone to transport secreted proteins. Its over-expression in resistant mosquitoes may suggest modulation of salivary gland cells due to the presence of the acetylcholinesterase resistance gene.

The present study also provided important data on the composition of the salivary gland of *C. quinquefasciatus*. Most of the identified proteins (57%) are involved in energy pathways, sugar feeding, protein folding, and the stress response. The remaining 43% are secreted proteins possibly associated with the blood meal. This finding confirms the work of Ribeiro *et al* [30] who reported a similar proportion of secreted proteins in *Culex* mosquitoes at the transcriptional level.

Previous proteomic studies showed that the secreted D7 proteins occurred in five short forms and two long forms in *Anopheles* mosquitoes [36] whereas two short forms and two long forms have been described in *Aedes* [37]. In *C. quinquefasciatus*, we found only three long forms of D7 (D7 Cluster12, D7 Cluster1, D7L3) which is inconsistent with previous results [30]. This may be explained by a failure to detect a small numbers of transcripts for short forms using a proteomic approach. Finally, salivary peptides (including those of the CWRP family) were identified by mass-spectrometry but the role of these proteins in *Culex* salivary glands requires further investigation.

Previous studies have demonstrated that the presence of the *Ace.1^R^* allele can affect several life history traits in mosquitoes [15], [17] Djogbénou *et al*, [38] recently reported a lack of *Ace.1^R^* homozygous resistance in *An. gambiae* populations from Burkina Faso, suggesting that the mutation may have a significant genetic cost. Effects of the *Ace.1^R^* allele on salivary protein down-expression might affect the fitness of homozygous resistant mosquitoes if their blood feeding success is significantly compromised by the presence of the mutation. Salivary proteins are injected into the skin to counteract the host's haemostatic reaction to the bite [39] and any modification in saliva composition could then modify the host's haemostatic response and thereby interfere with blood feeding success. Behavioural investigations using a video tracking system are needed to compare the flying, probing and biting behaviour of insecticide-resistant and susceptible mosquitoes. This will shed light on the impact of the modification of salivary proteins on the global fitness of resistant mosquitoes.

## Supporting Information

Table S1 **Salivary protein expression data.**
(DOC)
