# Supplementary material for: Differential Expression of Salivary Proteins between Susceptible and Insecticide-Resistant Mosquitoes of Culex quinquefasciatus
Source: PLoS One. 2011 Mar 23;6(3):e17496. doi: 10.1371/journal.pone.0017496 (PMC3063158; doi:10.1371/journal.pone.0017496)
Supplement: Table S1 — Salivary protein expression data. (DOC) [file pone.0017496.s001.doc]

**Table S1: Salivary protein expression data.**

This table contains all expression data for the identified proteins (with the corresponding q value and power) obtained from 2D-PAGE and Mass spectrometry between the insecticide-resistant strain SR and the susceptible strain SLAB.

| **Protein identification** |  | **Anova (p)** | **q Value** | **Power** |  | **Fold** |
| --- | --- | --- | --- | --- | --- | --- |
| **D7 clu12** |  | 2,97E-005 | 0,014906045 | 0,999793547 |  | 2,6 |
| **HSP83** |  | 1,39E-004 | 0,023425247 | 0,997215863 |  | 2,4 |
| **TPI** |  | 1,41E-004 | 0,023425247 | 0,997150465 |  | 2 |
| **D7 clu12** |  | 3,11E-004 | 0,038698104 | 0,991549402 |  | -2 |
| **Endo** |  | 4,32E-004 | 0,043323023 | 0,987274331 |  | 2 |
| D7 clu12 |  | 1,16E-003 | 0,082498809 | 0,962368552 |  | -1,9 |
| HSP70 |  | 1,56E-003 | 0,090947364 | 0,950001148 |  | 2 |
| NI |  | 3,47E-003 | 0,155328046 | 0,899912846 |  | 1,7 |
| NI |  | 7,49E-003 | 0,223795049 | 0,824089596 |  | 2 |
| EF1b, MLC, 30kSA Cq, SP |  | 8,43E-003 | 0,223795049 | 0,809797628 |  | 1,5 |
| DNAse 1 |  | 9,93E-003 | 0,223795049 | 0,788786359 |  | 1,6 |
| 20,2p |  | 9,99E-003 | 0,223795049 | 0,787866964 |  | 2,3 |
| NI |  | 1,63E-002 | 0,277178956 | 0,716404696 |  | 1,6 |
| NI |  | 1,91E-002 | 0,302980726 | 0,691057404 |  | -2,6 |
| NI |  | 2,54E-002 | 0,334314065 | 0,642341609 |  | -2,1 |
| NI |  | 2,54E-002 | 0,334314065 | 0,642232317 |  | -1,6 |
| MDH |  | 2,91E-002 | 0,341773294 | 0,6179672 |  | -1,4 |
| ALDH |  | 3,11E-002 | 0,363528783 | 0,605805883 |  | 1,3 |
| D7Clu1 |  | 3,63E-002 | 0,417082447 | 0,576880642 |  | 1,6 |
| Apy |  | 4,95E-002 | 0,417082447 | 0,517242386 |  | 1,9 |
|  |  |  |  |  |  |  |
| NI = not identified by mass spectrometry | |  |  |  |  |  |
